# Proteomic and *N*-glycomic comparison of synthetic and bovine whey proteins and their effect on human gut microbiomes *in vitro*

**DOI:** 10.1128/spectrum.00200-25

**Published:** 2025-06-26

**Authors:** Matthew Bolino, Hatice Duman, İzzet Avcı, Hacı Mehmet Kayili, Juli Petereit, Chandler Zundel, Bekir Salih, Sercan Karav, Steven A. Frese

**Affiliations:** 1 Department of Nutrition, University of Nevada, Reno 206786, Reno, Nevada, USA; 2 Department of Molecular Biology and Genetics, Çanakkale Onsekiz Mart University 52950, Çanakkale, Turkey; 3 Department of Chemistry, Faculty of Science, Hacettepe University 437145, Ankara, Turkey; 4 Department of Biomedical Engineering, Faculty of Engineering, Karabuk University 539327, Karabük, Turkey; 5 Nevada Bioinformatics Center, University of Nevada, Reno 6851, Reno, Nevada, USA; University of Nebraska-Lincoln, Lincoln, Nebraska, USA

**Keywords:** gut microbiome, fiber, *N*-glycan, whey, protein glycosylation

## Abstract

Advances in food production systems and customer acceptance have led to the commercial launch of dietary proteins produced via modern biotechnological approaches as alternatives to traditional agricultural sources. At the same time, a deeper understanding of how dietary components interact with the gut microbiome has highlighted the importance of understanding the nuances underpinning diet-microbiome interactions. Novel food proteins with distinct post-translational modifications resulting from their respective production systems have not been characterized, nor how they may differ from their traditionally produced counterparts. Here, we have characterized the protein composition and *N*-glycome of a yeast-synthesized and commercially available whey protein ingredient and compared this novel ingredient to whey protein isolate powder derived from bovine milk. Despite strong similarities in protein composition, we found that the *N*-glycome significantly differs between the two protein sources, reflecting the biosynthetic machinery of the production systems. Furthermore, the diversity of proteins found in yeast-synthesized whey protein were lower relative to bovine whey protein, despite both being predominantly β-lactoglobulin. Finally, to understand whether these differences in *N-*glycome profiles may affect the human gut microbiome, we compared these proteins in an *in vitro* fecal fermentation model. The two whey protein sources generated significant differences among three representative gut microbiomes *in vitro*, most likely due to differences in *N*-glycan composition and degradation by these representative microbial communities. This work highlights the need to understand how differences in novel biotechnological systems affect the bioactivity of synthesized proteins and how these differences impact the human gut microbiome.

**IMPORTANCE:**

Recent advances in food technology have led to the production of animal-free products from yeast that are traditionally derived from animals, such as milk proteins. These new processes raise important questions about the use of synthetic proteins as a replacement for traditionally sourced protein, especially in the context of the gut microbiome. Importantly, yeast produce *N*-glycans comprised primarily of mannose, while animals synthesize structurally and compositionally complex *N*-glycan structures. Given these differences, we characterized a new, yeast-derived whey protein ingredient and compared it to bovine whey protein. We found that yeast-derived whey protein differs in its impact on human gut microbiomes because of differences in *N*-glycan structures, despite similarity in protein composition. These findings raise important questions as to whether these differences in synthetic proteins lead to significant changes to the gut microbiome *in vivo*, and whether this may impact the utility of these novel ingredients.

## INTRODUCTION

There has been considerable interest in the development of novel protein sources to meet increasing global demands for dietary protein, especially those that meet consumer interests related to animal welfare, CO_2_ emissions, and land use practices (1). The interest in novel protein sources has resulted in significant commercial investment in developing viable protein sources that mimic or replace animal-derived food products. For example, popular plant-based milk alternatives lack many of the sensory and nutritional qualities of bovine milk (2, 3), and clinical recommendations caution against the substitution of plant-based milk alternatives for infants and children (4). Still, there has been a growing interest in developing a more robust alternative for bovine milk that is both functional and nutritionally more complete than currently available milk alternatives (5).

Recent developments in biosynthesis, fermentation, and heterologous gene expression technologies have resulted in the potential to produce a wide variety of dietary ingredients, including milk oligosaccharides (6), lipids (7), and protein (8). Biotechnological advances leveraging the power of microbial systems have also enabled the complex synthesis of large organic molecules and feedstocks for further organic molecule production (9, 10). More recently, this technology has been applied to the development of dietary ingredients derived from genes originating from other organisms to produce food proteins *in vitro*, the result of which is now commercially available in the United States (11).

Importantly, milk is a complex biological fluid that provides a sole source of nutrition for developing mammals and contains a wide variety of proteins, lipids, carbohydrates, and many bioactive compounds (12). The composition of milk has evolved to nourish, protect, and shape the development of the growing infant, and a full reconstruction of this nutritional and bioactive milieu is currently not feasible (11). However, where milk is a functional ingredient in other foods, it is possible that the primary organoleptic functions may be reconstructed without the associated complexity of the naturally occurring bioactive fluid. In the case of whey protein, whey is typically derived from cheese manufacturing, which retains milk caseins as the coagulated protein that forms cheese, while the whey proteins are removed during production and dried as a distinct ingredient. Compared to milk, whey is compositionally less complex and is predominantly made up of only a limited number of proteins, making it a more tractable system to replicate (11, 13).

While these novel technologies and ingredients may be conceptually attractive alternatives for consumers, there is little research characterizing these replacements for traditional food ingredients. For some ingredients, such as human milk oligosaccharides or synthetic organic chemical feedstocks, the chemical identity can be compared directly using techniques such as NMR. However, complex biomolecules such as proteins are subject to post-translational modifications, including *O-* and *N*-linked glycosylation, which requires additional characterization to describe the glycan structures bound to these proteins. These post-translational modifications have been the subject of intense research in recent years, and this research has shown that bioactive function, protein functionality, and interactions with the microbiome are affected by protein glycosylation or glycation (14–17). When considering the impact of these modifications on the gut microbiome, glycan structures can play an important role in shaping the ecological landscape of this community (16, 18, 19). For example, a novel lactoferrin produced in yeast via precision fermentation contains predominantly oligomannosidic *N-*glycans, while traditional bovine lactoferrin contains a mixture of oligomannosidic and complex structures (20), emphasizing that protein glycosylation is dictated by the organism, rather than the protein sequence.

Here, we examine the proteome and *N*-glycome of a commercially available yeast-synthesized whey protein ingredient and compare it to bovine whey protein isolate to determine how both protein identity and glycosylation patterns differ as a result of their respective biosynthetic origins. Using mass spectrometry to characterize these two protein sources, we were able to determine the protein composition and how *N*-glycosylation differs when proteins were produced in a yeast host, where their native *N-*glycosylation synthesis is distinct from mammalian *N*-glycosylation systems, and how these differences affect the human gut microbiome using an *in vitro* model system.

## MATERIALS AND METHODS

### Protein purification

Yeast-synthesized whey protein was purified from a commercially available food advertised to contain solely yeast-synthesized whey protein and was purchased at a local market. Yeast-synthesized whey samples (*n* = 5 replicates) were centrifuged at 4,000 RPM for 20 min to separate fat and centrifuge particulates. The de-fatted solution was then subjected to four rounds of ethanol precipitation by adding four volumes of 100% ice-cold ethanol, incubation at −20°C overnight, then followed by centrifugation at 4°C (4,000 RPM, 25 min) to remove residual sugars and other remaining contaminants. Bovine whey protein samples (*n* = 5 replicates) were obtained from commercial, powdered whey protein isolate, and purified in the same manner after suspension in water (20% wt/vol). The protein samples were subsequently aliquoted and dried at 30°C in a vacuum centrifuge. Purified protein was quantified using a Qubit BR Protein assay (ThermoFisher Scientific, Waltham, MA, USA) and then evaluated via denaturing SDS-PAGE in a 4%–15% acrylamide gel, stained with Coomassie (Bio-Safe Coomassie; Bio-Rad Laboratories, Inc, Hercules, CA, USA).

### Proteomic analysis

Purified protein extracts (*n* = 5 per protein source) were reduced, alkylated, and digested with a trypsin/Lys-C protease mixture using Thermo Scientific EasyPep Mini MS Sample prep kit (Cat #A40006). For LC-MS, peptides were trapped prior to separation on a 300 µm i.d. × 5 mm C18 PepMap 100 trap (Thermo Scientific, San Jose, CA, USA) and separated on a 50 cm uPAC C18 nano-LC column (PharmaFluidics, Ghent, Belgium) with a 15 µm tip using an UltiMate 3000 RSLCnano system (Thermo Scientific, San Jose, CA, USA). Mass spectral analysis was performed using an Orbitrap Eclipse mass spectrometer (Thermo Scientific, San Jose, CA, USA) using data-independent acquisition (DIA). Six gas phase fractions (GPF) of the biological sample pool were used to generate a reference library. The GPF acquisition used 4 m/z precursor isolation windows in a staggered pattern (GPF1 398.4-502.5 m/z, GPF2 498.5-602.5 m/z, GPF3 598.5-702.6 m/z, GPF4 698.6-802.6 m/z, GPF5 798.6-902.7 m/z, and GPF6 898.7-1002.7 m/z). Samples were analyzed on an identical gradient as the GPFs using a staggered 8 m/z window scheme over a mass range of 385–1,015 m/z. Library generation and data analysis were performed using Spectronaut software (Biognosys, Schlieren, Switzerland), and peptide mapping was repeated against a protein database that included all known bovine milk proteins (https://www.dallaslab.org/resources). Peptides mapping to porcine trypsin and a serine protease from *Achromobacter* were omitted from analysis as they were added during sample preparation.

### *N*-glycan profiling

Enzymatic *N-*linked deglycosylation of denatured protein samples was performed with PNGase F (1 Unit/μL) obtained from Promega (Madison, WI, USA). First, dried purified protein samples (1 mg) were dissolved in 50 µL of 2% SDS and denatured by incubation at 60°C. Denatured protein samples were then mixed with 2% NP-40 solution and 5× phosphate-buffered saline (PBS), and 1 U PNGase F was added and incubated at 37°C overnight. Finally, the samples were centrifuged, and supernatants were collected for further analysis. After enzymatic deglycosylation of protein samples, released *N*-glycans from each sample were labeled with a 2-AA tag. 50 µL of 2-AA tag (48 mg/mL^−1^ in DMSO/glacial acetic acid, 7:3, vol/vol) and 50 µL of 2-sodium cyanoborohydride (60 mg/mL in DMSO/glacial acetic acid, 7:3 wt/vol) were added to the released glycan samples (50 µL). Subsequently, the mixtures were incubated at 65°C for 2 hours. Purification of *N*-glycans was achieved by solid-phase extraction cartridges containing cellulose and porous graphitized carbon materials, as previously described (21). MALDI-TOF MS analysis of 2-AA labeled *N*-glycans from bovine and synthetic whey protein samples was carried out on a Bruker rapifleX MALDI Tissuetyper (Bruker Daltonik GmbH, Bremen, Germany) equipped with a SmartBeam 3D laser system. On the AnchorChip MALDI-target plate, the purified *N*-glycans (1 µL) were spotted and allowed to dry. Then, 1 µL of DHB matrix (5 mg/mL^−1^ in ACN/H_2_O, 1/1, vol/vol comprising 0.1% ortho-phosphoric acid) was added. The analysis included a 20 kV acceleration voltage, a 160 ns extraction delay, and the summation of 8,000 shots at 2,000 Hz for each spectrum. The mass range of 1,000–5,000 Da was used to produce all spectra using a random walk pattern in negative ion and reflectron mode. Data obtained by MALDI-TOF MS analysis were processed using Flex Analysis v.4.0 software (Bruker Daltonik Gmbh). Peaks of 2-AA labeled *N*-glycans were inserted into ProteinScape software, including the GlycoQuest algorithm (Bruker Daltonik GmbH, Bremen, Germany) for glycan identification. Total area normalization was used to determine the relative abundance of individual *N*-glycans (mass-intensity based). All experiments were performed with three technical replicates.

### Fecal sample collection and characterization

Fecal samples were collected from healthy individuals under the supervision of the University of Nevada, Reno Institutional Review Board (Approval #1751022), from which multiple aliquots were collected and stored at −80°C. DNA was extracted from these samples as previously described (22) using a ZymoBiomics DNA Miniprep kit (Zymo Research, Irvine, CA, USA) according to the manufacturer’s instructions, which included five rounds of bead beating for 1 minute, followed by incubation on ice for 1 minute. The resulting DNA was subjected to 16S rRNA sequencing of the V4 region using a previously described dual-indexed barcoding strategy (23) with recent modifications to the amplification sequences (24, 25). The V4 region of the 16S rRNA gene was amplified using the following primers 515F: AATGATACGGCGACCACCGAGATCTACACNNNNNNNN**TATGGTAATT***GT*GTGYCAGCMGCCGCGGTAA and 806R: CAAGCAGAAGACGGCATACGAGATNNNNNNNN**AGTCAGTCAG***CC*GGACTACNVGGGTWTCTAAT, which contained the modifications as described by Parada and Apprill. The primer sequences also contained an Illumina sequencing adapter (underlined), a unique eight-nucleotide barcode represented here as Ns, and a pad (bold) and linker sequence (italicized) to facilitate pooling and sequencing on an Illumina Miseq (23). Amplicons were generated in a HEPA-filtered laminar flow cabinet dedicated to PCR preparation and decontaminated before and after use. Kit and reaction controls were also included in downstream sequencing. Reactions were carried out using 200 nM of each primer, 0.5 mM added MgCl2, and GoTaq Master Mix (Promega; Madison, WI, USA) in 25 µL volumes with the following program in a MJ Research PTC-200 thermocycler: 94°C for 3 min, 25 cycles of 94°C for 45 s, 50°C for 60 s, and 72°C for 90 s, followed by a final extension at 72°C for 10 min. PCRs were pooled and purified with the High Pure PCR product purification kit (Roche Diagnostics; Mannheim, Germany), and 250 bp paired-end sequencing was performed on an Illumina MiSeq at the Idaho State University Molecular Research Core Facility.

Demultiplexed sequencing data were analyzed with QIIME2 (26). Reads were demultiplexed, trimmed to 200 bp, joined, denoised, and assigned to ASVs using DADA2 (27). Representative sequences were aligned with FastTree (28), and taxonomic assignments were assigned using the Silva database (v138, 99%; 29) with a feature classifier trained against the representative sequences. Distinct microbial communities were determined using the genus-level classification method described by Arumugan et al. (30) and available at https://enterotype.embl.de and in the supplementary data for this manuscript. After rarefaction analysis, samples were rarefied to 2,000 reads per sample strictly for diversity analyses, as recommended (31). The rarefaction analysis indicated that a sampling depth of 2,000 reads resulted in a plateau for α-diversity while retaining the greatest number of samples. ANCOM-BC (32) was performed using a frequency count table and was used to identify differentially abundant taxa as noted. Taxonomic data are reported at the family level as this has been shown to be the finest resolution level that maintains high confidence in taxonomic assignments (33).

### *In vitro* batch fermentation and analysis

As previously noted (34), fresh human fecal samples were frozen at −80°C within 4 hours of defecation as individual aliquots (~1 g each), along with a sample stored in DNA/RNA Shield (Zymo Research; Irvine, CA, USA) which was later used for 16S rRNA gene sequencing to identify the ecotype of the sample. Frozen aliquots were then diluted 1:10 (wt/vol) in cold sterile phosphate-buffered saline containing 15% glycerol to maintain viability, pooled in equal volumes, and frozen at −80°C. Aliquots were removed and thawed on ice prior to inoculation for *in vitro* experiments. Aliquots did not undergo any additional freeze-thaw cycles. Using an approach adapted from Aranda-Díaz et al. (35) and reported previously (34), each pooled community was inoculated (1% vol/vol) into 1 mL of a modified BHI medium in a deep-well 96-well plate as a control, or the same medium without the soluble starch and supplemented with bovine whey protein isolate (2% wt/vol) or yeast-synthesized whey protein (2% wt/vol) with four independent replicates per treatment per plate. The anaerobic culture medium was composed of BHI supplemented with 3.5 g/L of soluble starch, 0.3 g/L of L-cysteine HCl, 0.3 g/L of sodium thioglycolate, 1.5 mg/L of vitamin K1, and 0.3 mg/L of hemin and made anaerobic through mixed gas exchange in a Coy anaerobic chamber prior to fermentation. At inoculation, 100 µL of this culture was transferred to a 96-well plate and incubated at 37°C in a BioTek Epoch 2 96-well plate reader (Agilent Technologies; Santa Clara, CA, USA) housed within an anaerobic chamber with 1 minute of shaking prior to an OD_600 nm_ measurement every 30 minutes for 24 hours. The deep-well plate was sealed with sterile film, allowing for gas exchange, and incubated at 37°C in the same anaerobic chamber. The anaerobic gas used was 5% H_2_, 5% CO_2_, and 90% N_2_. An electronic oxygen monitor was housed within the anaerobic chamber to monitor anoxic conditions. After 12 and 24 hours, a replicate deep-well plate was removed from the anaerobic chamber, centrifuged at 4°C for 30 min (4,000 RPM), the supernatant was removed from the cell pellet into a new deep-well plate and stored at −80°C. DNA was extracted from pelleted cells using a ZymoBiomics 96 DNA kit (Zymo Research, Irvine, CA, USA) and subjected to 16S rRNA sequencing, as described above.

### Statistical methods

Statistical tests were performed in R (v. 4.2.2) (36). Relative protein abundances were compared between groups using a nonparametric Kruskal-Wallis test (37) using *ggpubr* (v. 0.4.0) and *rstatix* (v. 0.7.0) R packages (38, 39). The protein and *N*-glycan abundances were used to calculate the Bray-Curtis distance (40) between respective sample types using adonis in the *vegan* R package (v. 2.6.4) (41). Fecal community composition and abundance were assessed using the weighted UniFrac distance and compared using adonis and PERMANOVA tests in QIIME2 (v.2024.5). Figures were visualized using the *tidyverse*, *ggplot2* (v. 3.4.0), *ggpubr* (v. 0.4.0), and *viridis* (v. 0.6.2) R packages (38, 42–44).

## RESULTS

### Proteomic analysis and comparison of bovine and yeast-synthesized whey

**Fig 1:**
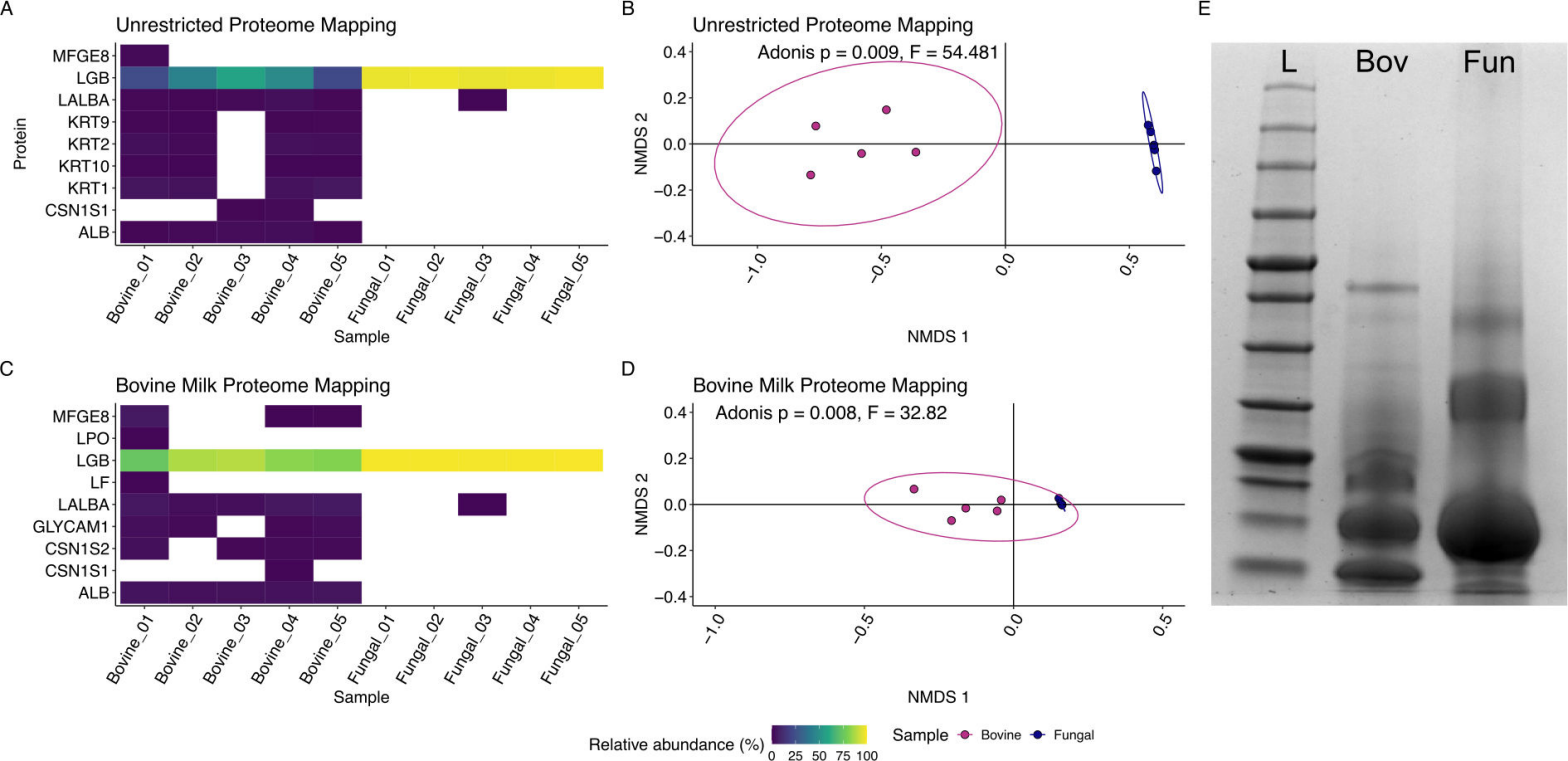
Characterization of bovine and yeast-synthesized whey by mass spectrometry identified similarities and differences. (**A**) Peptides mapped to an unrestricted protein database and were present at greater than 1% abundance were plotted and are colored by the relative abundance of the proteins. These results were also compared using a Bray-Curtis distance showing significant differences between the samples (B; ADONIS, *P* = 0.008), here visualized using nonmetric multidimensional scaling (NMDS). (**C**) In parallel, mapping peptides to a milk-specific protein database and (**D**) compared using the Bray-Curtis distance showing significant differences as well (ADONIS, *P* = 0.009), visualized by NMDS. Proteins used to digest input protein samples were also detected but were omitted from the analysis (porcine trypsin and a serine protease from *Achromobacter*). These findings were in agreement with SDS-PAGE of the same protein samples (**E**), showing a ladder (L), bovine whey (Bov),, and yeast-synthesized whey (Fun).

Mapping identified peptides produced from both the bovine and yeast-synthesized whey protein to either an unrestricted protein database or a bovine-milk-specific database produced a limited number of identified peptides, as expected. Among the most abundant proteins identified through the unrestricted protein database (>1% relative abundance), β-lactoglobulin, α-lactalbumin, albumin, and casein S1 were the most common milk proteins to which peptides from the bovine whey protein isolate could be mapped. Additional proteins identified included keratin proteins (KRT1, KRT2, KRT9, and KRT10), though at lower relative abundance (Table S1). When peptides from the bovine whey protein isolate were mapped to a protein database restricted to bovine milk proteins, the most abundant proteins (>1% relative abundance) to which peptides were mapped were β-lactoglobulin, α-lactalbumin, albumin, and casein S2. In addition, we identified GLYCAM1 (glycosylation-dependent cell adhesion molecule 1) and lactadherin in the bovine whey isolate protein source (Fig. 1; Table S1).

In contrast to the bovine whey protein isolate, which contained a mixture of proteins, both the unrestricted and the restricted milk protein database for peptide mapping identified the most abundant protein as β-lactoglobulin in the yeast-synthesized whey protein (>98% relative abundance; Fig. 1; Table 1; Table S1). These findings were in agreement with the analysis of these samples by SDS-PAGE, which found that in both sample types, β-lactoglobulin was the most abundant protein, with significantly less protein diversity in the yeast-derived whey protein sample (Fig. 1E). The relative abundance of β-lactoglobulin was 83% and 98% for the bovine and yeast-synthesized whey, respectively. We then compared the proteomes of the two samples using a Bray-Curtis dissimilarity metric and compared the protein composition of these samples using an Adonis test. Irrespective of the protein mapping database used for the peptide mapping, we found that the bovine and yeast-synthesized whey protein compositions were significantly different (*P* < 0.001; Fig. 1B and D). In the case of both protein samples, SDS-PAGE supported the findings by mass spectrometry (Fig. 1E).

**TABLE 1:** Relative abundance of proteins mapped using a milk-protein-specific database

Protein name	ProteinID	Associated gene	Organism	Bovine_01	Bovine_02	Bovine_03	Bovine_04	Bovine_05	Yeast_01	Yeast_02	Yeast_03	Yeast_04	Yeast_05
ABCG2	A7E3T8;B2D1N9;Q4GZT4	ABCG2	Bos taurus	ND^*a*^	0.00044109	0.00073052	0.0003074	ND	ND	ND	ND	ND	ND
ACTB	P60712	ACTB	Bos taurus	ND	ND	ND	ND	ND	ND	2.29E-05	1.02E-05	ND	ND
Perlipin 2	A1L5C2;Q9TUM6	PLIN2	Bos taurus	0.00025505	2.40E-05	9.84E-06	2.55E-05	ND	1.87E-06	3.62E-06	5.10E-05	ND	1.98E-06
AGP	Q5GN72	AGP	Bos taurus	ND	6.63E-05	0.00012206	ND	ND	7.88E-06	3.19E-06	5.68E-06	ND	4.53E-06
Albumin	P02769	ALB	Bos taurus	0.04079227	0.03230121	0.03367085	0.03813294	0.04194187	0.00096706	0.00070138	0.00106175	0.00077332	0.00061387
Zinc-α−2-glycoprotein	Q3ZCH5	AZGP1	Bos taurus	0.00360052	0.00237824	0.00206519	0.00265046	0.00111601	1.84E-05	2.85E-05	5.36E-05	2.66E-05	1.63E-05
Butyrophilin S1A1	P18892	BTN1A1	Bos taurus	0.00890999	0.00278628	0.00130795	0.0019386	0.00415791	2.97E-05	2.47E-05	2.86E-05	2.10E-05	1.75E-05
Cysteine rich secretory protein 2	Q3ZCL0	CRISP3	Bos taurus	0.00392798	0.00096722	0.00087098	0.0013248	0.00375732	2.76E-05	2.35E-05	2.08E-05	1.57E-05	2.36E-05
α-S1 casein	B5B3R8;P02662	CSN1S1	Bos taurus	0.00026342	0.00449284	0.00640146	0.01214973	0.00116513	0.00012612	0.00015519	0.00013509	0.00010071	0.00014964
α-S2 casein	P02663	CSN1S2	Bos taurus	0.02978757	0.00296628	0.01514065	0.01815394	0.02021645	0.00082907	0.00078446	0.00101651	0.00081509	0.00064958
β-casein	P02666	CSN2	Bos taurus	ND	ND	0.00019487	0.00016118	ND	6.29E-05	5.37E-05	3.37E-05	3.65E-05	3.59E-05
FABP3	P10790;P05413	FABP3	Bos taurus;Homo sapiens;	ND	ND	1.10E-06	2.08E-06	ND	ND	ND	ND	ND	ND
FGFBP1	Q9MZ06	FGFBP1	Bos taurus	ND	ND	ND	ND	ND	ND	4.53E-08	3.03E-08	ND	2.45E-08
Folate receptor α	P02702	FOLR1	Bos taurus	0.00014896	0.00025655	0.00034625	0.00044366	ND	3.39E-05	2.31E-05	2.88E-05	7.28E-06	2.13E-05
GLYCAM1	P80195	GLYCAM1	Bos taurus	0.02763987	0.01682093	0.00938096	0.01536089	0.02401186	0.00025314	0.00020107	0.00031989	0.0001282	0.00016104
α-Lactalbumin	P00711	LALBA	Bos taurus	0.0517926	0.02779232	0.02716334	0.05031422	0.05527366	0.00809745	0.00845758	0.01123701	0.00959802	0.00844608
Lactotransferrin	B9VPZ5;C7FE01;P24627	LF	Bos taurus	0.01053742	0.00701551	0.00638865	0.00763986	0.00928578	0.00011181	7.27E-05	8.82E-05	5.40E-05	4.74E-05
β-Lactoglobulin	P02754;Q9BDG3;Q9TRB9	LGB	Bos taurus	0.74188717	0.87205381	0.88381126	0.82065467	0.81171192	0.98896996	0.98895518	0.9852616	0.98800021	0.98946459
Lactoperoxidase	P80025	LPO	Bos taurus	0.01044199	0.00822976	0.00331652	0.00437202	0.00629889	5.57E-05	5.91E-05	7.04E-05	7.99E-05	5.00E-05
Lactadherin	Q95114	MFGE8	Bos taurus	0.05713906	0.00706318	0.00544668	0.0126597	0.01421478	0.00011337	0.00013912	0.00023316	0.00011686	6.80E-05
MUC15	Q8MI01	MUC15	Bos taurus	ND	ND	ND	ND	ND	3.78E-06	1.97E-06	5.95E-06	ND	ND
NPC2	P79345	NPC2	Bos taurus	0.00055765	0.00011031	0.0002746	0.00052403	0.00055287	8.16E-05	6.81E-05	7.94E-05	6.04E-05	5.88E-05
PIGR	P81265	PIGR	Bos taurus	0.00426959	0.00682484	0.00017922	0.0084789	0.00249049	9.53E-05	0.00010343	0.00011013	4.55E-05	8.35E-05
RNASE4	Q58DP6	RNASE4	Bos taurus	ND	0.00015689	6.02E-05	8.38E-05	ND	1.51E-05	7.46E-06	9.97E-06	ND	9.10E-06
Osteopontin	P31096	SPP1	Bos taurus	0.0007274	0.00251011	0.00160559	0.00029229	0.00015418	8.20E-05	8.76E-05	0.00010554	0.00010027	6.35E-05
Xanthine dehydogenase	P80457	XDH	Bos taurus	0.0073215	0.00474232	0.00151128	0.00432928	0.00365087	1.62E-05	2.23E-05	3.30E-05	2.03E-05	1.36E-05

^*a*^ ND, not detected.

### *N*-glycan analysis and comparison to bovine whey

**Fig 2:**
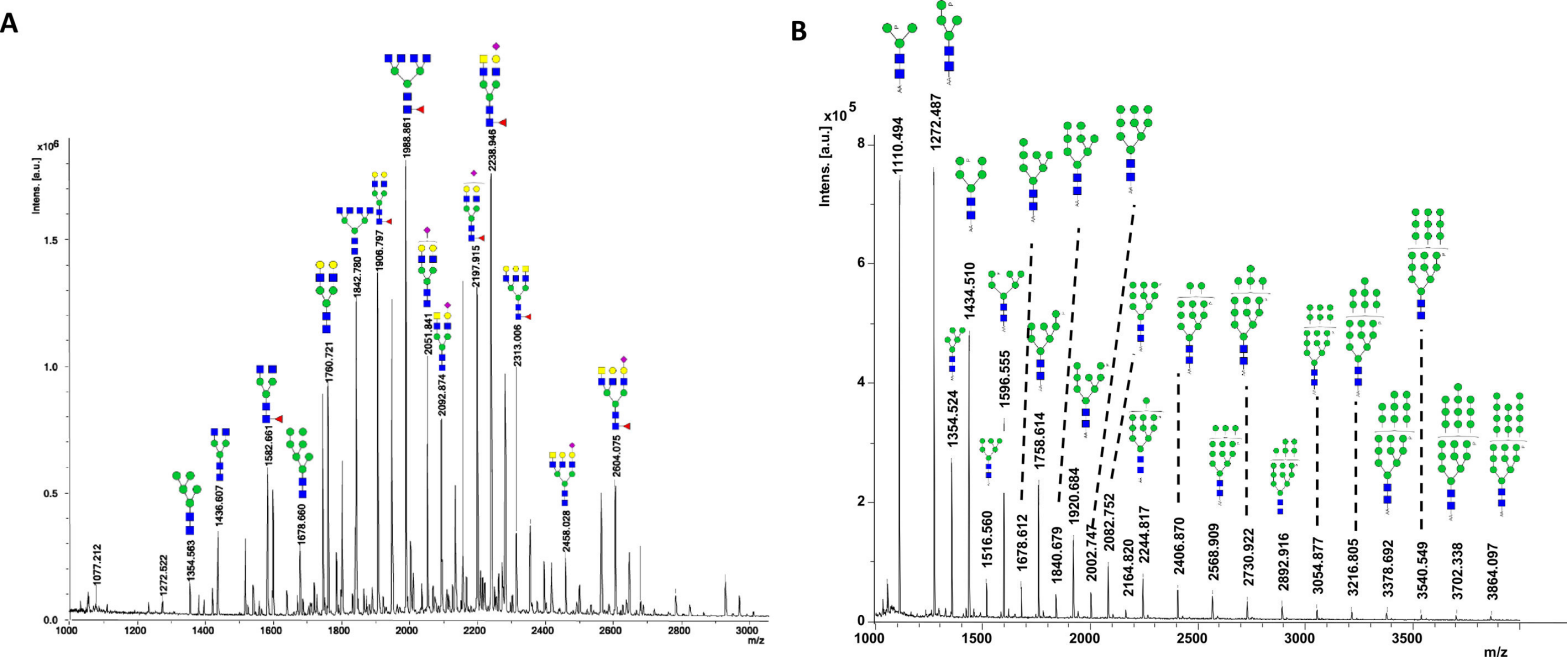
Identification of *N*-glycans identified in bovine whey protein and yeast-derived whey protein. Proteins released from bovine whey protein isolate and yeast-synthesized whey protein by PNGase-F were identified by mass spectrometry. (**A**) The most abundant *N*-glycans from bovine whey identified by mass spectra are shown. These glycans were characterized as being high mannose, complex, and hybrid, and fucosylated complex and hybrid *N*-glycans were identified. Six of the fifteen most abundant *N*-glycans in bovine whey protein were identified as sialylated, while the remaining nine were considered neutral *N*-glycans. (**B**) In contrast to the bovine whey, all of the yeast-synthesized whey *N*-glycan structures were identified as neutral, high mannose *N*-glycans without fucosylation.

*N*-glycans were released from protein samples using enzymatic deglycosylation with PNGase F and analyzed by matrix-assisted laser desorption/ionization time-of-flight coupled with mass spectrometry (MALDI-TOF MS) as described in Materials and Methods. The resulting mass spectra (Fig. 2) were used to identify distinct glycan structures, which included 78 structures from the bovine whey protein sample and 22 total structures from the yeast-synthesized whey proteins. *N-*glycans are broadly characterized to be high mannose, hybrid, or complex *N*-glycans, depending on their composition and can be further distinguished by whether they are decorated with sialic acid, yielding neutral or acidic *N*-glycan structures. Here, we found that the *N*-glycome of the bovine whey protein isolate contained 78 distinct structures, which included 9 neutral high mannose *N*-glycans; 4 neutral hybrid *N*-glycans; 2 acidic hybrid *N*-glycans; 31 neutral complex *N*-glycans; and 32 acidic complex *N*-glycans. By contrast, the *N*-glycome of the yeast-synthesized whey protein contained 22 structures, all containing mannose and phosphorylated mannose-type glycans. Of these glycans, 10 structures were shared between the two sample types, while 12 were unique to the yeast-synthesized whey and 67 were unique to bovine whey.

**Fig 3:**
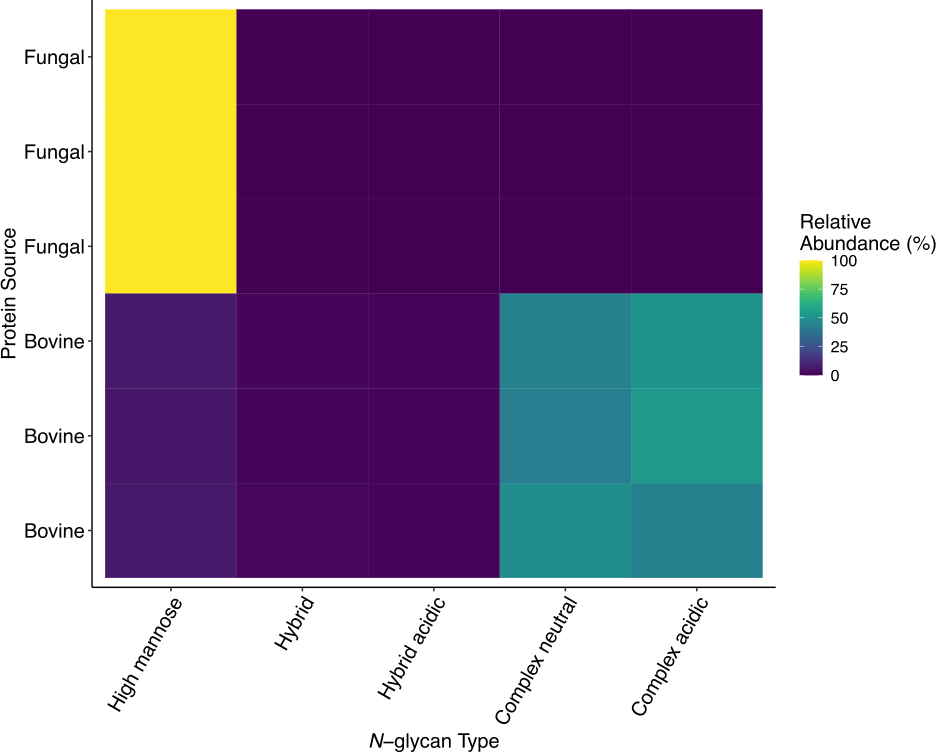
*N*-glycan class relative abundance among bovine and protein samples. *N*-glycans released from bovine whey protein isolate and yeast-synthesized whey protein samples by PNGase-F were identified by mass spectrometry and classified by composition to either neutral or acidic (containing sialic acid) and high mannose, complex, or hybrid structure. The relative abundance of each of these classes of *N*-glycans is colored by abundance across the tested samples.

The composition and characterization of these *N*-glycans are presented in Table 2. The relative abundance of neutral high mannose *N*-glycans as a fraction of the total glycome was higher in the yeast-synthesized whey protein sample, while there were more neutral and acidic complex *N-*glycans in the bovine whey protein glycome (Fig. 3).

**TABLE 2:** *N*-glycans identified across whey protein isolate samples

Source	Composition	m/z meas.	m/z calc.	Int.
Bovine	Hex6HexNAc3NeuAc1dHex1-AA	2156.8908	2156.7644	223,037
Bovine	Hex6HexNAc3NeuAc1-AA	2010.8054	2010.7065	164,583
Bovine	Hex6HexNAc3-AA	1719.6909	1719.6111	130,722
Bovine	Hex6HexNAc3dHex1-AA	1865.7635	1865.6690	106,057
Bovine	Hex8HexNAc2-AA	1840.7289	1840.6374	323,076
Bovine	Hex9HexNAc2-AA	2002.8043	2002.6902	290,291
Bovine	Hex6HexNAc2-AA	1516.6035	1516.5317	289,777
Bovine	Hex7HexNAc2-AA	1678.6599	1678.5846	241,294
Bovine	Hex5HexNAc2-AA	1354.5631	1354.4789	132,476
Bovine	Hex5HexNAc4NeuAc1dHex1-AA	2197.9155	2197.7910	1,299,175
Bovine	Hex5HexNAc4NeuAc1-AA	2051.8415	2051.7331	635,677
Bovine	Hex4HexNAc5NeuAc1dHex1-AA	2238.9456	2238.8175	1,779,069
Bovine	Hex3HexNAc6NeuAc1dHex1-AA	2279.9775	2279.8441	926,524
Bovine	Hex4HexNAc5NeuAc1-AA	2092.8742	2092.7596	790,109
Bovine	Hex5HexNAc6NeuAc1dHex1-AA	2604.0750	2603.9497	514,264
Bovine	Hex5HexNAc6NeuAc1-AA	2458.0580	2457.8918	227,479
Bovine	Hex6HexNAc5NeuAc1-AA	2417.0008	2416.8653	221,323
Bovine	Hex7HexNAc6NeuAc1dHex1-AA	2928.0898	2928.0554	142,383
Bovine	Hex4HexNAc4NeuAc1dHex1-AA	2035.8421	2035.7382	121,896
Bovine	Hex4HexNAc4NeuAc1-AA	1889.7714	1889.6803	114,081
Bovine	Hex3HexNAc4NeuAc1-AA	1727.6955	1727.6274	87,990
Bovine	Hex4HexNAc3NeuAc1dHex1-AA	1832.7373	1832.6588	85,996
Bovine	Hex4HexNAc3NeuAc1-AA	1686.6679	1686.6009	71,519
Bovine	Hex5HexNAc3NeuAc1-AA	1848.7320	1848.6537	68,600
Bovine	Hex5HexNAc5NeuAc1dHex1-AA	2400.9918	2400.8704	55,623
Bovine	Hex3HexNAc6dHex1-AA	1988.8613	1988.7487	1,689,244
Bovine	Hex5HexNAc4dHex1-AA	1906.7973	1906.6956	1,198,851
Bovine	Hex4HexNAc5dHex1-AA	1947.8298	1947.7221	1,140,178
Bovine	Hex3HexNAc6-AA	1842.7803	1842.6908	1,013,288
Bovine	Hex5HexNAc4-AA	1760.7206	1760.6377	822,363
Bovine	Hex4HexNAc4dHex1-AA	1744.7239	1744.6427	790,146
Bovine	Hex4HexNAc5-AA	1801.7503	1801.6642	551,858
Bovine	Hex3HexNAc4dHex1-AA	1582.6612	1582.5899	531,023
Bovine	Hex4HexNAc4-AA	1598.6551	1598.5848	437,118
Bovine	Hex4HexNAc7dHex1-AA	2354.0327	2353.8809	366,818
Bovine	Hex5HexNAc6dHex1-AA	2313.0064	2312.8543	337,768
Bovine	Hex3HexNAc4-AA	1436.6072	1436.5320	295,048
Bovine	Hex3HexNAc5dHex1-AA	1785.7564	1785.6693	241,281
Bovine	Hex4HexNAc3dHex1-AA	1541.6326	1541.5634	126,245
Bovine	Hex5HexNAc5dHex1-AA	2109.8984	2109.7749	106,119
Bovine	Hex3HexNAc5-AA	1639.6845	1639.6114	104,066
Bovine	Hex3HexNAc3dHex1-AA	1379.5919	1379.5105	91,628
Bovine	Hex6HexNAc7dHex1-AA	2678.1153	2677.9865	67,239
Bovine	Hex5HexNAc3-AA	1557.6296	1557.5583	65,797
Yeast	Hex6HexNAc3NeuAc1dHex1-AA	2156.7719	2156.7644	102,412
Yeast	Hex6HexNAc3NeuAc1-AA	2010.7015	2010.7065	76,055
Yeast	Hex6HexNAc3-AA	1719.5926	1719.6111	58,990
Yeast	Hex6HexNAc3dHex1-AA	1865.6582	1865.6690	50,950
Yeast	Hex4HexNAc2P1-AA	1272.4400	1272.3924	1.99E + 06
Yeast	Hex5HexNAc2P1-AA	1434.4570	1434.4450	1,603,341
Yeast	Hex6HexNAc2P1-AA	1596.4600	1596.4981	1.35E + 06
Yeast	Hex8HexNAc2P1-AA	1920.6990	1920.6037	598,056
Yeast	Hex9HexNAc2P1-AA	2083.0620	2082.6565	411,019
Yeast	Hex9HexNAc2-AA	2002.8930	2002.6902	394,699
Yeast	Hex10HexNAc2P1-AA	2245.6380	2244.7094	350,372
Yeast	Hex8HexNAc2-AA	1840.6262	1840.6374	155,217
Yeast	Hex10HexNAc2-AA	2165.3560	2164.7430	138,251
Yeast	Hex4HexNAc2-AA	1192.4290	1192.4261	97,429
Yeast	Hex5HexNAc2-AA	1354.482	1354.4789	64,817
Yeast	Hex4HexNAc5NeuAc1dHex1-AA	2238.8308	2238.8175	888,851
Yeast	Hex5HexNAc4NeuAc1dHex1-AA	2197.8012	2197.7910	620,814
Yeast	Hex3HexNAc6NeuAc1dHex1-AA	2279.8579	2279.8441	445,397
Yeast	Hex4HexNAc5NeuAc1-AA	2092.7646	2092.7596	344,777
Yeast	Hex5HexNAc4NeuAc1-AA	2051.7332	2051.7331	274,257
Yeast	Hex6HexNAc5NeuAc1dHex1-AA	2562.9202	2562.9232	218,697
Yeast	Hex6HexNAc5NeuAc1-AA	2416.8742	2416.8653	96,942
Yeast	Hex6HexNAc4NeuAc1-AA	2213.7958	2213.7859	74,204
Yeast	Hex7HexNAc6NeuAc1dHex1-AA	2927.9376	2928.0554	66,544
Yeast	Hex4HexNAc4NeuAc1dHex1-AA	2035.7342	2035.7382	58,592
Yeast	Hex5HexNAc4NeuGc1-AA	2067.7363	2067.7280	56,925
Yeast	Hex4HexNAc4NeuAc1-AA	1889.6661	1889.6803	52,014
Yeast	Hex3HexNAc4NeuAc1-AA	1727.6037	1727.6274	43,190
Yeast	Hex4HexNAc3NeuAc1dHex1-AA	1832.6443	1832.6588	42,845
Yeast	Hex7HexNAc6NeuAc1-AA	2781.9404	2781.9975	41,988
Yeast	Hex5HexNAc5NeuAc1-AA	2254.8326	2254.8124	41,424
Yeast	Hex5HexNAc3NeuAc1-AA	1848.6101	1848.6537	40,540
Yeast	Hex4HexNAc3NeuAc1-AA	1686.5723	1686.6009	35,340
Yeast	Hex3HexNAc6dHex1-AA	1988.7541	1988.7487	797,976
Yeast	Hex3HexNAc4S1-AA	1516.4880	1516.5317	634,753
Yeast	Hex5HexNAc4dHex1-AA	1906.6922	1906.6956	567,343
Yeast	Hex4HexNAc5dHex1-AA	1947.7241	1947.7221	514,273
Yeast	Hex3HexNAc6-AA	1842.6790	1842.6908	420,562
Yeast	Hex4HexNAc4dHex1-AA	1744.6264	1744.6427	370,546
Yeast	Hex5HexNAc4-AA	1760.6214	1760.6377	338,208
Yeast	Hex3HexNAc4dHex1-AA	1582.5704	1582.5899	260,014
Yeast	Hex4HexNAc5-AA	1801.6515	1801.6642	222,464
Yeast	Hex4HexNAc4-AA	1598.5631	1598.5848	197,992
Yeast	Hex4HexNAc7dHex1-AA	2353.9070	2353.8809	158,445
Yeast	Hex5HexNAc6dHex1-AA	2312.8770	2312.8543	141,884
Yeast	Hex3HexNAc4-AA	1436.5162	1436.5320	133,243
Yeast	Hex4HexNAc4S1-AA	1678.5613	1678.5417	112,297
Yeast	Hex3HexNAc5dHex1-AA	1785.6580	1785.6693	109,301
Yeast	Hex3HexNAc8dHex1-AA	2394.9317	2394.9074	95,566
Yeast	Hex6HexNAc5dHex1-AA	2271.8483	2271.8278	93,025
Yeast	Hex4HexNAc7-AA	2207.8359	2207.8230	70,107
Yeast	Hex5HexNAc6-AA	2166.8106	2166.7964	64,991
Yeast	Hex4HexNAc3dHex1-AA	1541.5404	1541.5634	56,386
Yeast	Hex5HexNAc5dHex1-AA	2109.7754	2109.7749	50,661
Yeast	Hex3HexNAc3dHex1-AA	1379.5041	1379.5105	46,507
Yeast	Hex3HexNAc5-AA	1639.5918	1639.6114	44,854
Yeast	Hex6HexNAc5-AA	2125.7809	2125.7699	44,433
Yeast	Hex4HexNAc3-AA	1395.4562	1395.5055	42,668
Yeast	Hex7HexNAc6dHex1-AA	2636.9481	2636.9600	35,075
Yeast	Hex6HexNAc7dHex1-AA	2677.9637	2677.9865	32,025
Yeast	Hex5HexNAc3-AA	1557.5273	1557.5583	31,955

### Microbial communities remain distinct at 12 and 24 hours of *in vitro* fermentation

**Fig 4:**
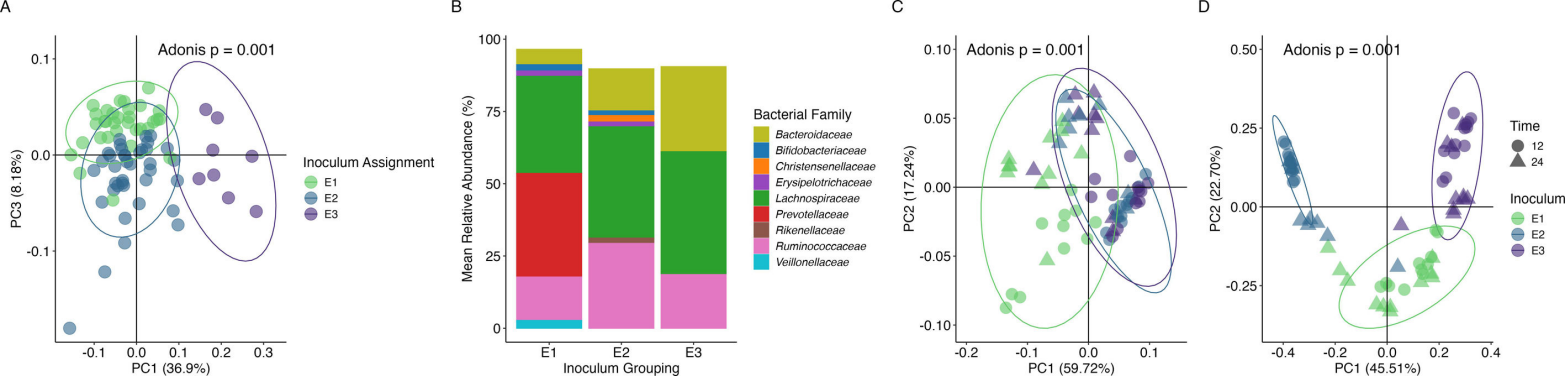
Microbial communities remain distinct at 12 and 24 hours. (**A**) Microbiome compositions of input fecal samples. (**B**) Family-level stack plots of the starting microbial communities demonstrate distinct microbiota compositional state (Adonis *P* = 0.001), and after fermentation, distinct compositions remain at 12 and 24 hours (*P* = 0.001) when assessed by (**C**) weighted UniFrac and (**D**) Bray-Curtis dissimilarity measures. Bacterial families with a mean relative abundance less than 1.5% are omitted from the stacked bar plot.

We first evaluated the fidelity of the *in vitro* system to faithfully recapitulate the distinct compositions detected in the input microbial communities. First, 16S rRNA gene sequencing identified three significantly distinct microbial community clusters (ADONIS, *P* = 0.001, Fig. 4A) among fecal samples. We then followed up with pairwise PERMANOVA comparisons and observed significant differences between each microbial community cluster (FDR-adjusted *P* = 0.001). These clusters were identified as previously described (30), and six samples from each cluster were randomly selected for pooling to create the three taxonomically distinct microbial communities tested here (Fig. 4B) and in our previous work (45). First, we tested whether these three representative microbial communities remained distinct during *in vitro* fermentation or whether the *in vitro* method collapsed community diversity to common features. We compared the three distinct communities grown on our soluble starch control at 12 and 24 hours post-inoculation. Using the weighted UniFrac distance metric, we found that the three distinct communities from the inocula remained distinct at 12 and 24 hours (ADONIS, *P* = 0.001, Fig. 4C), which was corroborated by the non-phylogenetic Bray Curtis distance metric at 12 and 24 hours (ADONIS, *P* = 0.001, Fig. 4D). Post hoc PERMANOVA comparisons using the Bray-Curtis metric showed that each community remained distinct at 12 and 24 hours (FDR-adjusted *P* < 0.05).

### Impact of glycoprotein source on human gut microbial communities

To understand whether differences in *N-*glycomes between the bovine and yeast-synthesized whey protein influenced microbial composition, we exposed each one of our starting microbial communities to one of the following conditions as sole carbon sources: (i) yeast-synthesized whey (ii), bovine whey isolate, or (iii) soluble starch, as a control. We then assessed the beta diversity of each microbial community when grown under different conditions at 12 and 24 hours using the Bray-Curtis distance metric. Interestingly, only microbial community E1 had significant differences in beta diversity between the conditions at both 12 and 24 hours (ADONIS, *P* < 0.05); however, no significant differences in beta diversity were detected in either microbial community E2 or E3 at either 12 or 24 hours (ADONIS, *P* > 0.05). Weighted UniFrac detected significant differences in beta diversity between conditions within microbial communities E1 and E3 at 12 hours (ADONIS, *P* < 0.05), and between conditions within all microbial communities at 24 hours (ADONIS, *P* < 0.05). Additional pairwise comparisons within microbial communities between conditions at 12 or 24 hours found that microbial community E1 grown on bovine whey protein was significantly different in terms of community composition at 24 hours when compared to the control and yeast-synthesized whey protein (FDR-adjusted *P* < 0.05) when assessed by the Bray-Curtis distance metric, while E2 grown with yeast-synthesized whey protein was significantly different from the bovine and control groups at 12 hours. Microbial community E3, by contrast, showed no significant differences after correction for multiple comparisons (FDR-adjusted *P* > 0.05). Pairwise comparisons using the weighted UniFrac revealed no significant differences in community composition between any two conditions within any microbial community at any timepoint.

**Fig 5:**
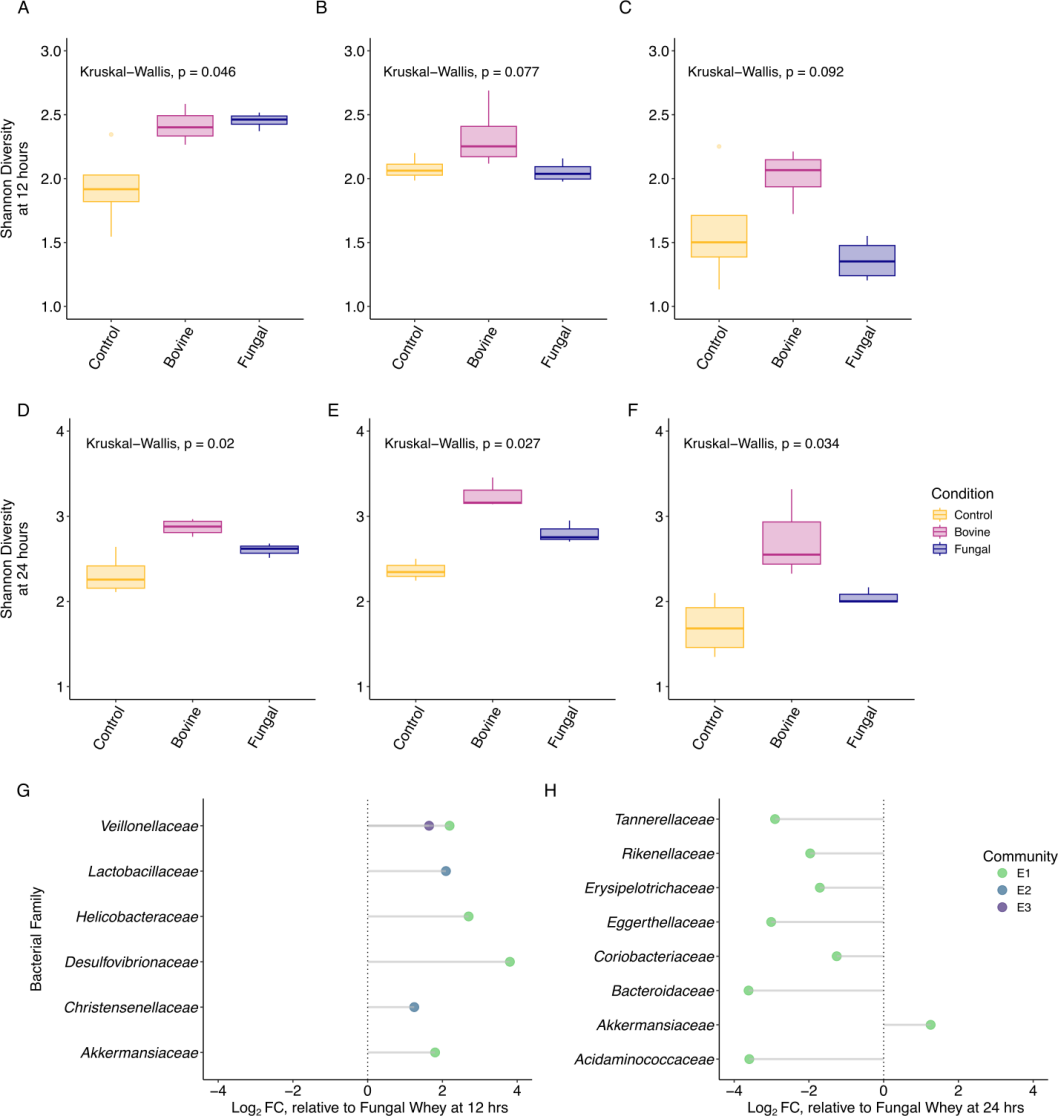
Alpha diversity and family-level taxa differ across microbial communities and substrates. Microbial communities (columns) show distinct responses to substrates (conditions) in terms of Shannon diversity at 12 and 24 hours (A–C, D–F, respectively), and ANCOM-BC determined glycoprotein sources enrich taxa at the family level in a substrate and community-specific manner at 12 and 24 hours (G and H, respectively).

To test whether distinct *N*-glycan structures affect diversity among these *in vitro* microbial communities, we compared the Shannon diversity of each microbial community when grown on either bovine or yeast-synthesized whey glycoprotein at 12 or 24 hours. At 12 hours, communities incubated with bovine whey protein were significantly more diverse than the control (FDR-adjusted *P* < 0.05, Fig. 5A through C), but other comparisons were not significantly different (FDR-adjusted *P* > 0.05). At 24 hours, all three communities incubated with bovine whey protein were significantly more diverse than the control (FDR-adjusted *P* < 0.001) and the communities incubated with the yeast-synthesized whey protein (FDR-adjusted *P* < 0.05, Fig. 5D through F). While the communities incubated with yeast-synthesized whey protein trended higher in terms of diversity than the control, these differences were not significant at 12 or 24 hours (Fig. 5A through F).

Finally, we performed Analysis of Compositions of Microbiomes with Bias Correction (ANCOM-BC) to identify differentially abundant taxa at the family level within each microbial community in response to either bovine or yeast-synthesized whey glycoproteins at 12 and 24 hours of fermentation. All three starting microbial communities displayed significant enrichment in family-level taxa at 12 hours of fermentation when grown on bovine whey relative to yeast-synthesized whey (q < 0.05; Fig. 5G), suggesting that the differences in *N-*glycan architecture between glycoprotein sources were driving family-level enrichment. Within microbial community E1 at 12 hours of fermentation, bovine whey samples were significantly enriched for *Veillonellaceae*, *Helicobacteraceae*, *Desulfovibrionaceae*, and *Akkermansiaceae* in relation to the yeast-synthesized whey. Within microbial community E2 at 12 hours of fermentation, bovine whey samples were significantly enriched for *Lactobacteraceae* and *Christensenellaceae*. Lastly, within microbial community E3 at 12 hours, the only family that was differentially abundant was *Veillonellaceae*, which was significantly enriched on bovine relative to the yeast-synthesized whey. Interestingly, microbial communities E2 and E3 did not exhibit any significant differences in family enrichment at 24 hours of fermentation; however, microbial community E1 contained eight families that significantly differed in abundance between the glycoprotein substrates. Within microbial community E1 at 24 hours of fermentation, bovine whey only significantly enriched for *Akkermansiaceae*, while yeast-synthesized whey enriched for *Tannerellaceae*, *Rikenellaceae*, *Erysipelotrichaceae*, *Eggerthellaceae*, *Coriobacteriaceae*, *Bacteroidaceae*, and *Acidaminococcaceae* (Fig. 5H). Overall, no family was significantly enriched across all three microbial communities on either glycoprotein at 12 and 24 hours of fermentation, suggesting community-specific responses to the glycoproteins. Interestingly, *Akkermansiaceae* within microbial community E1 was the only family significantly enriched across 12 and 24 hours of fermentation. No other family remained significantly enriched across both timepoints on either glycoprotein substrate (Fig. S1).

## DISCUSSION

Advances in biosynthetic technologies have rapidly reshaped the development of food ingredients, with an aim to meet consumer perceptions about animal agriculture and a desire for improved efficiency in food production (1). However, despite technological advancements, these novel technologies cannot yet fully recapitulate the biological systems that have historically produced these food ingredients, even if the synthesis of individual constituents is technologically feasible (11). Here, we evaluated the composition of a novel yeast-synthesized whey protein ingredient in terms of its proteomic composition and *N*-glycome in comparison to whey protein isolate derived from bovine milk. Using mass spectrometry, we characterized the proteomic composition and *N*-glycome of two distinct whey protein sources.

While bovine-derived whey and the yeast-synthesized whey ingredient are both principally composed of β-lactoglobulin, the yeast-synthesized whey protein was almost entirely β-lactoglobulin, notably higher in relative abundance compared to bovine-derived whey. The remaining proteins detected in the yeast-derived whey could have originated from the production organism. By contrast, the bovine whey protein isolate was composed of more proteins, present at greater than 1% of the total composition, and exhibited greater total diversity relative to the yeast-derived whey. Consistent with previous work on bovine whey, these other proteins included α-lactalbumin, albumin, and casein S1 (13, 46). While these protein samples were significantly different in terms of their composition (Fig. 1), the overwhelming predominance of β-lactoglobulin in both samples (83% vs. 98%) suggests that the functional properties of both ingredients may be largely comparable (13), though we did not examine the effects of processing on glycation, nor the functional effects this may have, in the present work (14).

However, in contrast to the protein content, the *N*-glycome of these whey protein samples was remarkably distinct. Although β-lactoglobulin, not considered an *N*-glycosylated protein (47), was the predominant protein in the yeast-derived whey protein sample, we detected a wide variety of *N*-glycans in the yeast-synthesized whey protein sample that included a large number of unique structures absent in the bovine whey protein (Fig. 3; Table 2). All *N*-glycans synthesized within eukaryotic organisms share a core structure of two 4Glc*N*Acβ1 sugars and a single mannose stemming into two branched mannose monomers (48). Depending on the organism, the final *N*-glycan is extended from this core through distinct biosynthetic pathways, resulting in a variety of *N*-glycan structures that reflect the machinery in the host cell, such as those found between animal and plant sources (49). For example, yeast-derived *N*-glycans are structurally distinct from the hybrid or complex *N*-glycans that are often reported for bovine milk protein *N*-glycans, and the heterologous expression of mammalian proteins in yeast requires significant modification to the yeast glycosylation machinery to mimic the *N*-glycosylation of mammals (50, 51). Furthermore, it is important to note that *N*-glycans affect multiple characteristics of glycoproteins such as conformation, solubility, antigenicity, activity, and recognition by glycan-binding proteins (15, 48, 52, 53). While β-lactoglobulin is not thought to be *N*-glycosylated (47), other synthetic food proteins with bioactive function that are *N-*glycosylated are likely to suffer functional deficits if glycosylation is not addressed in their manufacture.

Finally, there is existing evidence that *N*-glycans can serve as substrates for members of the gut microbiome, and specific adaptations for *N*-glycan utilization among gut microbes have also been described (16, 17, 54, 55). We found that despite similar protein compositions between bovine and yeast-synthesized whey glycoproteins, fermentation of these glycoproteins after 12 hours revealed family-level differential abundance across all three microbial communities’ derived from human fecal microbiota. In addition, all three microbial communities displayed significantly higher alpha diversity when grown on bovine whey compared to the yeast-synthesized whey after 24 hours. Given our findings relating to *N-*glycan composition, we hypothesize that the differential abundance at 12 hours and differences in alpha diversity at 24 hours between glycoproteins were driven by the distinct glycosylation patterns observed, as well as the glycan fermentation capacity of each microbial community.

One limitation of the present study is that we have employed an *in vitro* model, though we have demonstrated that this model is robust, sensitive to dose-dependent effects of similar food ingredients, and representative of human gut enterotypes (45). Furthermore, while these ingredients were not pre-treated with proteases to simulate gastric digestion, future work could test the effect of protease digestion on these substrates. However, we hypothesize that as the proteome of both protein samples was comparable, any effect on the results presented here could be difficult to identify. Further testing will be necessary to characterize how *N-*glycan utilization differs among gut microbiome compositions, whether distinct genetic repertoires are found among these microbiome compositional types, and to identify which functional pathways are responsible for these differences.

In conclusion, we found that while the protein composition of a novel, yeast-synthesized whey protein ingredient is distinct from bovine-derived whey protein isolate, the most distinguishing characteristic was the *N*-glycome of the respective protein samples, which was dependent on their origin. Thus, while we examined two commercially available dietary protein ingredients with comparable functionality, we found deeper distinctions between them that may affect bioactive functionality in other systems that use similar biosynthetic machinery, as well as their respective impacts on the human gut microbiome.

## Data Availability

Proteomics and MALDI-TOF MS spectra are available in the Dryad data repository (https://doi.org/10.5061/dryad.hmgqnk9qz). 16S rRNA amplicon sequencing data have been deposited in the NCBI SRA under BioProject PRJNA984714.

## SUPPLEMENTAL MATERIAL

Supplemental materials spectrum.00200-25-s0001.docx Fig. S1 and Table S1.

ASM does not own the copyrights to Supplemental Material that may be linked to, or accessed through, an article. The authors have granted ASM a non-exclusive, world-wide license to publish the Supplemental Material files. Please contact the corresponding author directly for reuse.
