# Supplementary material for: Proteomic and N-glycomic comparison of synthetic and bovine whey proteins and their effect on human gut microbiomes in vitro
Source: Microbiol Spectr. 2025 Jun 26;13(8):e00200-25. doi: 10.1128/spectrum.00200-25 (PMC12323674; doi:10.1128/spectrum.00200-25)
Supplement: Supplemental materials — Fig. S1 and Table S1. [file spectrum.00200-25-s0001.docx]

**Supplementary Materials.**

**
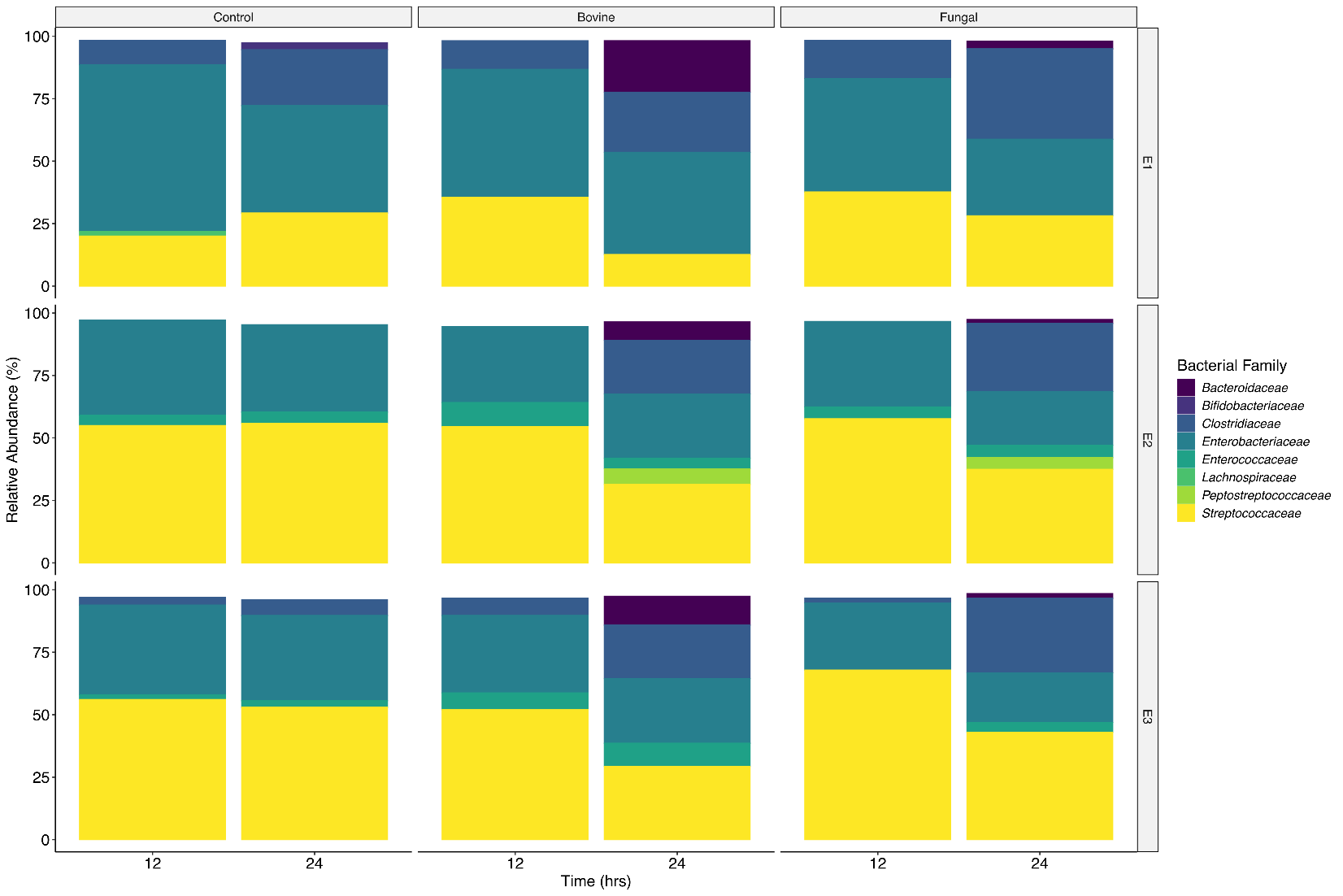
**

**Supplementary Figure 1.** Stacked bar plots showing the relative abundance of family-level taxa within each microbial community after fermentation of respective glycoprotein or starch substrates at 12- and 24-hours. Bacterial families with a mean relative abundance less than 1.5% are omitted.

**Table S1.** Relative abundance of proteins mapped using an unrestricted proteome database (Spectronaut).

| ProteinGroups | Genes | Organisms | Bovine_01 | Bovine_02 | Bovine_03 | Bovine_04 | Bovine_05 | Yeast_01 | Yeast_02 | Yeast_03 | Yeast_04 | Yeast_05 |
| --- | --- | --- | --- | --- | --- | --- | --- | --- | --- | --- | --- | --- |
| Q5GN72 | A1GP | Bos taurus | NA | 3.34E-05 | 8.02E-05 | NA | NA | 7.71E-06 | 3.14E-06 | 5.57E-06 | NA | 4.49E-06 |
| A7E3T8;B2D1N9;Q4GZT4 | ABCG2 | Bos taurus | NA | 0.00022246 | 0.00047991 | 0.00017876 | NA | NA | NA | NA | NA | NA |
| P60712 | ACTB | Bos taurus | NA | NA | NA | NA | NA | NA | 2.25E-05 | 9.99E-06 | NA | NA |
| Q3ZCH5 | AZGP1 | Bos taurus | 0.00113321 | 0.00119943 | 0.00135674 | 0.00154128 | 0.00030714 | 1.80E-05 | 2.81E-05 | 5.26E-05 | 2.62E-05 | 1.62E-05 |
| P18892 | BTN1A1 | Bos taurus | 0.00280428 | 0.00140522 | 0.00085926 | 0.00112733 | 0.00114433 | 2.91E-05 | 2.44E-05 | 2.80E-05 | 2.07E-05 | 1.74E-05 |
| Q3ZCL0 | CRISP3 | Bos taurus | 0.00123627 | 0.0004878 | 0.00057219 | 0.0007704 | 0.00103408 | 2.70E-05 | 2.32E-05 | 2.04E-05 | 1.55E-05 | 2.34E-05 |
| P02666 | CSN2 | Bos taurus | NA | NA | 0.00012802 | 9.37E-05 | NA | 6.17E-05 | 5.30E-05 | 3.30E-05 | 3.60E-05 | 3.56E-05 |
| P10790 | FABP3 | Bos taurus | NA | NA | 7.20E-07 | 1.21E-06 | NA | NA | NA | NA | NA | NA |
| Q9MZ06 | FGFBP1 | Bos taurus | NA | NA | NA | NA | NA | NA | 4.47E-08 | 2.97E-08 | NA | 2.43E-08 |
| P02702 | FOLR1 | Bos taurus | 4.69E-05 | 0.00012939 | 0.00022747 | 0.000258 | NA | 3.32E-05 | 2.28E-05 | 2.82E-05 | 7.16E-06 | 2.11E-05 |
| P80195 | GLYCAM1 | Bos taurus | 0.00869921 | 0.00848337 | 0.00616285 | 0.0089326 | 0.00660847 | 0.00024793 | 0.00019814 | 0.00031362 | 0.00012616 | 0.00015964 |
| P04264 | KRT1 | Homo sapiens | 0.04144703 | 0.03418386 | 0.00977919 | 0.03852316 | 0.04921692 | 0.00241079 | 0.00103517 | 0.00249946 | 0.00135173 | 0.00214943 |
| P13645 | KRT10 | Homo sapiens | 0.01440723 | 0.01202681 | 0.00378521 | 0.01174265 | 0.01216158 | 0.00053828 | 0.00018422 | 0.00056666 | 0.00025069 | 0.00033302 |
| O77727 | KRT15 | Ovis aries | 0.00037046 | 0.00014645 | NA | 6.55E-05 | 0.00164391 | 1.40E-05 | 2.76E-05 | 2.16E-05 | 8.81E-06 | 1.26E-05 |
| P35908 | KRT2 | Homo sapiens | 0.02931456 | 0.02003348 | 0.00678848 | 0.03055995 | 0.02836981 | 0.00141059 | 0.00053426 | 0.00135216 | 0.00066464 | 0.00094735 |
| Q9NSB2 | KRT84 | Homo sapiens | NA | 4.05E-05 | NA | 3.24E-05 | 0.00131182 | 1.87E-05 | 9.56E-06 | 2.47E-05 | 1.79E-06 | 2.82E-05 |
| P35527 | KRT9 | Homo sapiens | 0.01400109 | 0.01026889 | 0.00252213 | 0.01318662 | 0.01610428 | 0.00054188 | 0.00026773 | 0.00070848 | 0.00050635 | 0.0007174 |
| P00711 | LALBA | Bos taurus | 0.01630091 | 0.01401662 | 0.01784505 | 0.02925853 | 0.01521224 | 0.00793087 | 0.00833438 | 0.01101677 | 0.00944573 | 0.00837254 |
| B9VPZ5;C7FE01;P24627 | LF | Bos taurus | 0.00331649 | 0.00353816 | 0.00419705 | 0.0044427 | 0.0025556 | 0.00010951 | 7.16E-05 | 8.65E-05 | 5.32E-05 | 4.70E-05 |
| P80025 | LPO | Bos taurus | 0.00328645 | 0.00415055 | 0.0021788 | 0.0025424 | 0.00173356 | 5.46E-05 | 5.82E-05 | 6.91E-05 | 7.86E-05 | 4.95E-05 |
| Q95114 | MFGE8 | Bos taurus | 0.01798362 | 0.00356221 | 0.00357821 | 0.00736182 | 0.00391215 | 0.00011104 | 0.00013709 | 0.00022859 | 0.00011501 | 6.74E-05 |
| Q8MI01 | MUC15 | Bos taurus | NA | NA | NA | NA | NA | 3.70E-06 | 1.95E-06 | 5.83E-06 | NA | NA |
| P79345 | NPC2 | Bos taurus | 0.00017551 | 5.56E-05 | 0.0001804 | 0.00030473 | 0.00015216 | 7.99E-05 | 6.71E-05 | 7.78E-05 | 5.95E-05 | 5.83E-05 |
| P81265 | PIGR | Bos taurus | 0.00134379 | 0.003442 | 0.00011774 | 0.00493062 | 0.00068543 | 9.33E-05 | 0.00010192 | 0.00010797 | 4.48E-05 | 8.28E-05 |
| A1L5C2;Q9TUM6 | PLIN2 | Bos taurus | 8.03E-05 | 1.21E-05 | 6.46E-06 | 1.49E-05 | NA | 1.83E-06 | 3.56E-06 | 5.00E-05 | NA | 1.96E-06 |
| Q58DP6 | RNASE4 | Bos taurus | NA | 7.91E-05 | 3.96E-05 | 4.87E-05 | NA | 1.48E-05 | 7.35E-06 | 9.77E-06 | NA | 9.02E-06 |
| P62979 | RPS27A | Homo sapiens | 0.00057067 | 0.0001303 | 0.00029184 | 0.00071874 | 0.000189 | 0.00072517 | 0.00059232 | 0.00057069 | 0.00081207 | 0.0006723 |
| P15636 | Ser-protease | Achromobacter lyticus | 0.07876314 | 0.05548734 | 0.03916406 | 0.05317584 | 0.08894653 | 0.00323082 | 0.00152018 | 0.00235546 | 0.00263091 | 0.00089375 |
| P31096 | SPP1 | Bos taurus | 0.00022894 | 0.00126594 | 0.0010548 | 0.00016997 | 4.24E-05 | 8.03E-05 | 8.63E-05 | 0.00010347 | 9.87E-05 | 6.29E-05 |
| P00761 | trypsin | Sus scrofa | 0.50543923 | 0.36080691 | 0.27824498 | 0.2670041 | 0.52520614 | 0.01168166 | 0.01039641 | 0.01150075 | 0.00964075 | 0.00295347 |
| P80457 | XDH | Bos taurus | 0.00230433 | 0.00239172 | 0.00099284 | 0.00251755 | 0.00100478 | 1.59E-05 | 2.20E-05 | 3.24E-05 | 2.00E-05 | 1.35E-05 |
| P02769 | ALB | Bos taurus | 0.013791 | 0.01883179 | 0.02459063 | 0.02564982 | 0.01317627 | 0.00094717 | 0.00069116 | 0.00104094 | 0.00076105 | 0.00060852 |
| B5B3R8;P02662;P02663 | CSN1S1 | Bos taurus | 0.00945808 | 0.00376189 | 0.01415217 | 0.01762207 | 0.00588457 | 0.00093554 | 0.00092596 | 0.00112902 | 0.00090127 | 0.00079227 |
| Q9TRB9;Q9BDG3;P02754 | LGB | Bos taurus | 0.23349735 | 0.43980661 | 0.58062305 | 0.47722397 | 0.22339681 | 0.96862494 | 0.97454859 | 0.96595048 | 0.97232287 | 0.98084879 |
